# Supplementary material for: Gut uropathogen abundance is a risk factor for development of bacteriuria and urinary tract infection
Source: Nat Commun. 2019 Dec 4;10:5521. doi: 10.1038/s41467-019-13467-w (PMC6893017; doi:10.1038/s41467-019-13467-w)
Supplement: Supplementary file 1 — Supplementary Information [file 41467_2019_13467_MOESM1_ESM.pdf]

Supplementary Information

Gut Uropathogen Abundance is a Risk Factor for Development of Bacteriuria and Urinary Tract Infection

Matthew Magruder[1], Nidal A Sholi[1], Catherine Gong[1], Lisa Zhang[1], Emmanuel Edusei[1], Jennifer Huang[1], Shady Albakry[1], Michael J Satlin[2], Lars F Westblade[2,3], Carl Crawford[4], Darshana Dadhania[1,5], Michelle Lubetzky[1,5], Ying Taur[6], Eric Littman[6], Lilan Ling[6], Philip Burnham[7], Iwijn De Vlaminck[7], Eric Pamer[6], Manikkam Suthanthiran[1,5], John Richard Lee*[1,5]

[1] Division of Nephrology and Hypertension, Department of Medicine, Weill Cornell Medicine, New York, NY

[2] Division of Infectious Diseases, Department of Medicine, Weill Cornell Medicine, New York, NY

[3] Department of Pathology and Laboratory Medicine, Weill Cornell Medicine, New York, NY

[4] Division of Gastroenterology, Department of Medicine, Weill Cornell Medicine, New York, NY

[5] Department of Transplantation Medicine, New York Presbyterian Hospital – Weill Cornell Medical Center, New York, NY

[6] Infectious Disease Services, Department of Medicine, Memorial Sloan Kettering Cancer Center, New York, NY

[7] Department of Biomedical Engineering, Cornell University, Ithaca, NY

* Address correspondence to: Dr. John Richard Lee, jrl2002@med.cornell.edu

Supplementary Table 1. Characteristics in the Bacteriuria Group and the No Bacteriuria Group.

Characteristic	Bacteriuria Group (N=102) N (%) / Median	No Bacteriuria Group (N=66) N (%) / Median	P value
**Age**	**57**	**52**	**0.05**
**Female Gender**	**60 (59%)**	**16 (24%)**	**1.5E-05**
African American Race	31 (30%)	13 (20%)	0.15
History of DM	32 (31%)	17 (26%)	0.49
Prior Kidney Transplantation	12 (12%)	12 (18%)	0.27
Cause of End Stage Renal Disease			
DM or DM/HTN	31 (30%)	17 (26%)	0.60
HTN	19 (19%)	8 (12%)	0.29
Other	52 (51%)	41 (62%)	0.20
cPRA status (≥80%)	9 (9%)	4 (6%)	0.57
Liver/Kidney Dual Transplantation	1 (1%)	1 (2%)	0.99
Kidney/Pancreas Dual Transplantation	1 (1%)	2 (3%)	0.56
Deceased Donor Transplantation	33 (32%)	16 (24%)	0.30
Ureteral Stent Placement	102 (100%)	66 (100%)	0.99
Prolonged Foley Catheter	3 (3%)	0 (0%)	0.28
Delayed Graft Function	20 (20%)	8 (12%)	0.29
Induction Therapy			
Anti-thymocyte Globulin	74 (73%)	54 (82%)	0.20
Basiliximab	28 (27%)	11 (17%)	0.13
None	0 (0%)	1 (2%)	0.39
Preoperative Antibiotic Prophylaxis			
Cefazolin	83 (81%)	56 (85%)	0.68
Vancomycin	14 (14%)	6 (9%)	0.47
Other	5 (5%)	4 (6%)	0.74
Pneumocystis jiroveci Prophylaxis			

Trimethoprim/Sulfamethoxazole	98 (96%)	61 (92%)	0.32
Dapsone	2 (2%)	0 (0%)	0.52
Atovaquone	2 (2%)	5 (8%)	0.11
Tacrolimus Maintenance	100 (98%)	66 (100%)	0.52
Prednisone Maintenance	30 (29%)	15 (23%)	0.38

The distribution of continuous variables was compared using the Wilcoxon rank sum test and the distribution of categorical variables was compared using a Fisher's exact test. Delayed graft function is defined as the need for hemodialysis within the first 7 days after kidney transplantation. Prolonged foley catheter is defined as the need for a foley catheter beyond 7 days or intermittent self-catherization. Abbreviations: DM, diabetes mellitus; HTN, hypertension; cPRA, calculated panel reactive antibodies. Source data are provided as a source data file.

Supplementary Table 2. Bacteriuria Culture Data in the Cohort.

Type	Number of Patients	Number of Events
Coagulase-negative staphylococci[a]	44	86
Escherichia coli	36	126
Enterococcus spp[b]	36	106
Klebsiella pneumoniae	20	46
Streptococcus spp[c]	19	32
Pseudomonas spp[d]	8	24
Corynebacterium spp, not *Corynebacterium urealyticum*	8	8
Proteus mirabalis	3	5
Citrobacter freundii	3	3
Enterobacter cloacae	2	9
Pantoea aglomerans	2	3
Lactobacillus spp.	2	2
Serratia marcescens	1	2
Brevundimonas diminuta	1	1
Raoutella ornithinolytica	1	1
Stenotrophomonas maltophilia	1	1

[a] Among the 86 cultures, there were 78 coagulase-negative staphylococci, 7 coagulase-negative staphylococci, not *Staphylococcus Saprophyticus*, and 1 *Staphylococcus epidermis.*

[b] Among the 106 cultures, there were 90 *Enterococcus faecalis*, 10 *Enterococcus faecium*, 3 *Enterococcus avium*, and 3 *Enterococcus gallinarum.*

[c] Among the 32 cultures, there were 21 viridans Group streptococci and 11 *Streptococcus agalactiae.*

[d] Among the 24 cultures, there were 21 *Pseudomonas aeruginosa*, 2 *Pseudomonas putida*, and 1 *Pseudomonas fluorescens.*

Source data are provided as a source data file.

Supplementary Table 3. Gut Microbiota Abundance and Future Development of *Escherichia* UTI.

	Univariate Analysis		Multivariate Analysis	
Characteristic	HR (95% CI)	P value	HR (95% CI)	P value
Age, Years	1.0 (1.0 - 1.0)	0.94		
**Female Gender**	**8.6 (2.5 - 29.1)**	**5.30E-04**	**8.4 (2.5 - 28.4)**	**0.001**
African American Race	1.1 (0.4 - 2.8)	0.83		
Diabetes Mellitus	1.1 (0.4 - 2.6)	0.89		
Prior Kidney Transplant	0.6 (0.1 - 2.4)	0.43		
Cause of ESRD - DM	0.9 (0.4 - 2.2)	0.81		
Cause of ESRD - HTN	0.2 (0.0 - 1.7)	0.15		
PRA ≥ 80%	0.5 (0.1 - 3.9)	0.53		
Decreased Donor Transplantation	1.4 (0.6 - 3.5)	0.40		
Delayed Graft Function	0.5 (0.1 - 2.1)	0.35		
Cefazolin Preoperative Abx	0.7 (0.3 - 19)	0.49		
TMP-SMX PCP Prophylaxis	*	*		
Anti-thymocyte Globulin Induction	2.2 (0.6 - 7.4)	0.21		
Prednisone Maintenance	0.8 (0.3 - 2.1)	0.61		
**1% *Escherichia* Relative Abundance**	**2.9 (1.3 - 6.8)**	**0.01**	**2.8 (1.2 - 6.5)**	**0.02**

A Cox Proportion Hazard Model was used to assess the relationship between gut microbial abundance and future development of *Escherichia* UTI. A 1% relative gut abundance of *Escherichia* was assessed as a time-dependent covariate. The hazard ratio (HR) with 95% confidence intervals (CI) is reported with the associated *P* value. Univariate analysis was performed with all of the characteristics. Characteristics that were significantly associated with *Escherichia* UTI (P <0.10) were further analyzed in the multivariate analysis. In bold text are the characteristics associated with future development of *Escherichia* UTI. Cause of ESRD is ordinal data and the reference for the HR is Cause of ESRD – Other. * TMP-SMX PCP prophylaxis characteristics was unable to be analyzed because there

were no cases of *Escherichia* UTIs in the non TMP-SMX PCPR Prophylaxis Group. Abbreviations: DM, diabetes mellitus; HR: Hazard Ratio; HTN – hypertension; PRA, panel reactive antibodies; TMP-SMX, Trimethoprim-Sulfamethoxazole; PCP: *Pneumocystis jiroveci.* Source data are provided as a source data file.

Supplementary Table 4. Gut Microbiota Abundance and Future Development of *Enterococcus* UTI.

Risk Factors for *Enterococcus* UTI

Characteristic	Univariate Analysis HR (95% CI)	Univariate Analysis P value	Multivariate Analysis HR (95% CI)	Multivariate Analysis P value
Age, Years	1.0 (0.9 - 1.0)	0.37		
**Female Gender**	**3.2 (1.0 - 10.1)**	**0.05**	**3.0 (0.9 - 9.5)**	**0.07**
African American Race	1.7 (0.6 - 5.1)	0.33	0.2 (0.0 - 1.5)	0.11
**Diabetes Mellitus**	**0.2 (0.0 - 1.3)**	**0.09**		
Prior Kidney Transplant	0.4 (0.1 - 3.4)	0.44		
Cause of ESRD - DM	0.2 (0.0 - 1.6)	0.14		
Cause of ESRD - HTN	1.7 (0.5 - 5.5)	0.38		
PRA ≥ 80%	0.9 (0.1 - 7.2)	0.95		
Decreased Donor Transplantation	1.0 (0.3 - 3.1)	0.98		
Delayed Graft Function	0.4 (0.0 - 2.9)	0.35		
Cefazolin Preoperative Abx	2.8 (0.4 - 21.4)	0.32		
Bactrim PCP Prophylaxis	0.8 (0.1 - 5.8)	0.79		
Anti-thymocyte Globulin Induction	1.9 (0.4 - 8.5)	0.40		
Prednisone Maintenance	0.8 (0.2 - 2.8)	0.70		
1% Enterococcus Relative Abundance	1.8 (0.6 - 5.6)	0.28		

A Cox Proportion Hazard Model was used to assess the relationship between gut microbial abundance and future development of *Enterococcus* UTI. A 1% relative gut abundance of *Enterococcus* was assessed as a time-dependent covariate. The hazard ratio (HR) with 95% confidence intervals (CI) is reported with the associated *P* value. Univariate analysis was performed with all of the characteristics. Characteristics that were significantly associated with *Enterococcus* UTI (P <0.10) were further analyzed in the

multivariate analysis. In bold text are the characteristics associated with future development of *Enterococcus* UTI. Cause of ESRD is ordinal data and the reference for the HR is Cause of ESRD – Other. Abbreviations: DM, diabetes mellitus; HR: Hazard Ratio; HTN – hypertension; PRA, panel reactive antibodies; TMP-SMX, Trimethoprim-Sulfamethoxazole; PCP: *Pneumocystis jiroveci.* Source data are provided as a source data file.

Supplementary Table 5. Single Nucleotide Variant Differences Per 100 Base Pairs Between Consensus *E. coli* Strains.

	Subject 83 Stool 2	Subject 83 Stool 1	Subject 54 Stool 2	Subject 54 Stool 1	Subject 54 Urine 2	Subject 77 Stool	Subject 90 Stool	Subject 36 Stool	Subject 36 Urine	Subject 41 Stool	Subject 7 Stool	Subject 7 Urine	Subject 41 Urine	Subject 106 Urine	Subject 106 Stool	Subject 128 Stool	Subject 3 Stool	Subject 48 Stool	Subject 165 Urine	Subject 165 Stool
Subject 83 Stool 2	0.00	0.03	0.09	0.09	1.43	1.61	1.64	1.43	1.46	1.43	1.49	2.04	1.52	2.98	2.98	2.75	2.99	2.54	3.58	2.51
Subject 83 Stool 1	0.03	0.00	0.06	0.06	1.40	1.64	1.61	1.40	1.49	1.46	1.52	2.01	1.49	2.95	2.95	2.72	2.96	2.54	3.55	2.48
Subject 54 Stool 2	0.09	0.06	0.00	0.00	1.34	1.58	1.55	1.35	1.43	1.52	1.58	2.07	1.55	3.01	3.01	2.78	3.02	2.48	3.61	2.54
Subject 54 Stool 1	0.09	0.06	0.00	0.00	1.34	1.58	1.55	1.35	1.43	1.52	1.58	2.07	1.55	3.01	3.01	2.78	3.02	2.48	3.61	2.54
Subject 54 Urine 2	1.43	1.40	**1.34**	**1.34**	0.00	2.36	2.33	2.13	2.22	2.19	2.25	2.74	2.22	3.87	3.87	3.60	3.87	3.54	4.62	3.54
Subject 77 Stool	1.61	1.64	1.58	1.58	2.36	0.00	0.03	0.74	0.77	1.23	1.29	1.84	1.32	2.54	2.54	2.31	2.66	2.22	3.37	2.31
Subject 90 Stool	1.64	1.61	1.55	1.55	2.33	0.03	0.00	0.71	0.80	1.26	1.32	1.81	1.29	2.51	2.51	2.28	2.63	2.22	3.34	2.28
Subject 36 Stool	1.43	1.40	1.35	1.35	2.13	0.74	0.71	0.00	0.09	1.17	1.23	1.72	1.20	2.60	2.60	2.36	2.72	2.31	3.43	2.25
Subject 36 Urine	1.46	1.49	1.43	1.43	2.22	0.77	0.80	**0.09**	0.00	1.20	1.20	1.81	1.29	2.69	2.69	2.45	2.81	2.36	3.52	2.34
Subject 41 Stool	1.43	1.46	1.52	1.52	2.19	1.23	1.26	1.17	1.20	0.00	0.06	0.60	0.09	2.19	2.19	2.13	2.31	2.25	3.31	2.25
Subject 7 Stool	1.49	1.52	1.58	1.58	2.25	1.29	1.32	1.23	1.20	0.06	0.00	0.60	0.09	2.19	2.19	2.19	2.36	2.31	3.37	2.31
Subject 7 Urine	2.04	2.01	2.07	2.07	2.74	1.84	1.81	1.72	1.81	0.60	0.60	0.00	**0.57**	2.69	2.69	2.69	2.83	2.83	3.75	2.81
Subject 41 Urine	1.52	1.49	1.55	1.55	2.22	1.32	1.29	1.20	1.29	**0.09**	**0.09**	0.57	0.00	2.16	2.16	2.16	2.34	2.31	3.34	2.28
Subject 106 Urine	2.98	2.95	3.01	3.01	3.87	2.54	2.51	2.60	2.69	2.19	2.19	2.69	2.16	0.00	**0.00**	1.14	1.55	2.13	3.42	2.28
Subject 106 Stool	2.98	2.95	3.01	3.01	3.87	2.54	2.51	2.60	2.69	2.19	2.19	2.69	2.16	0.00	0.00	1.14	1.55	2.13	3.42	2.28
Subject 128 Stool	2.75	2.72	2.78	2.78	3.60	2.31	2.28	2.36	2.45	2.13	2.19	2.69	2.16	1.14	1.14	0.00	1.14	2.07	3.22	2.07
Subject 3 Stool	2.99	2.96	3.02	3.02	3.87	2.66	2.63	2.72	2.81	2.31	2.36	2.83	2.34	1.55	1.55	1.14	0.00	2.48	3.63	2.48
Subject 48 Stool	2.54	2.54	2.48	2.48	3.54	2.22	2.22	2.31	2.36	2.25	2.31	2.83	2.31	2.13	2.13	2.07	2.48	0.00	2.86	1.66
Subject 165 Urine	3.58	3.55	3.61	3.61	4.62	3.37	3.34	3.43	3.52	3.31	3.37	3.75	3.34	3.42	3.42	3.22	3.63	2.86	0.00	**1.17**
Subject 165 Stool	2.51	2.48	2.54	2.54	3.54	2.31	2.28	2.25	2.34	2.25	2.31	2.81	2.28	2.28	2.28	2.07	2.48	1.66	1.17	0.00

The 20 consensus *E. coli* strains are listed on both the first column and the first row. The distance between two strains (single nucleotide variant differences per 100 base pairs) is listed in each of the boxes. Highlighted in yellow are the urine strains and in bold are the closest consensus strains by distance. Source data are provided as a source data file.

Supplementary Table 6. Single Nucleotide Variant Differences Per 100 Base Pairs Between Consensus *E. faecalis* Strains.

	Subject 83 Stool 1	Subject 7 Stool	Subject 83 Stool 2	Subject 3 Stool	Subject 4 Urine	Subject 4 Stool	Subject 54 Urine 2	Subject 54 Stool 1	Subject 120 Stool	Subject 54 Stool 2
Subject 83 Stool 1	0.00	0.23	0.19	1.05	1.28	0.84	1.62	0.91	0.91	0.96
Subject 7 Stool	0.23	0.00	0.42	1.26	1.48	1.04	1.82	1.11	1.12	1.17
Subject 83 Stool 2	0.19	0.42	0.00	1.06	1.21	0.77	1.48	0.76	0.77	0.82
Subject 3 Stool	1.05	1.26	1.06	0.00	1.53	1.08	1.90	1.17	1.18	1.21
Subject 4 Urine	1.28	1.48	1.21	1.53	0.00	**0.49**	1.57	0.86	0.87	0.91
Subject 4 Stool	0.84	1.04	0.77	1.08	0.49	0.00	1.08	0.38	0.38	0.43
Subject 54 Urine 2	1.62	1.82	1.48	1.90	1.57	1.08	0.00	**0.73**	**0.73**	0.78
Subject 54 Stool 1	0.91	1.11	0.76	1.17	0.86	0.38	0.73	0.00	0.01	0.05
Subject 120 Stool	0.91	1.12	0.77	1.18	0.87	0.38	0.73	0.01	0.00	0.06
Subject 54 Stool 2	0.96	1.17	0.82	1.21	0.91	0.43	0.78	0.05	0.06	0.00

The 10 consensus *E. faecalis* strains are listed on both the first column and the first row. The distance between two strains (single nucleotide variant differences per 100 base pairs) is listed in each of the boxes. Highlighted in yellow are the urine strains and in bold are the closest consensus strains by distance. Source data are provided as a source data file.

Supplementary Table 7. Single Nucleotide Variant Differences Per 100 Base Pairs Between Consensus *E. faecium* Strains.

	Subject 54 Stool 2	Subject 54 Stool 1	Subject 54 Urine 1	Subject 83 Stool 2	Subject 83 Stool 1
Subject 54 Stool 2	0.00	0.00	0.48	0.15	4.68
Subject 54 Stool 1	0.00	0.00	0.48	0.15	4.68
**Subject 54 Urine 1**	**0.48**	**0.48**	0.00	0.61	5.15
Subject 83 Stool 2	0.15	0.15	0.61	0.00	4.60
Subject 83 Stool 1	4.68	4.68	5.15	4.60	0.00

The 5 consensus *E. faecium* strains are listed on both the first column and the first row. The distance between two strains (single nucleotide variant differences per 100 base pairs) is listed in each of the boxes. Highlighted in yellow are the urine strains and in bold are the closest consensus strains by distance. Source data are provided as a source data file.

Supplementary Table 8

Antibiotic	N
Beta-lactams	133
Penicillins	
Piperacillin-tazobactam	36
Amoxicillin	16
Ampicillin	7
Penicillin V	2
Cephalosporins	
Ceftriaxone	13
Cefpodoxime	12
Cefazolin	9
Cephalexin	7
Cefepime	2
Ceftolozane/tazobactam	1
Cefadroxil	1
Carbapenems	
Meropenem	15
Ertapenem	6
Monobactam	
Aztreonam	6
Fluoroquinolones	78
Levofloxacin	50
Ciprofloxacin	28
Other	
Vancomycin (Intravenous)	20
Metronidazole	9
Isoniazid	8
Linezolid	8
Vancomycin (Oral)	7
Azithromycin	6
Trimethoprim	6
Nitrofurantoin	5
Doxycycline	3
Clindamycin	2
Amikacin	1

Daptomycin	1
Erythromycin	1
Fosfomycin	1

Antibiotic Usage in the Cohort. Antibiotic courses in the first 3 months after kidney transplantation are listed in the cohort by classes. Source data are provided as a source data file.

Supplementary Table 9

Risk Factors for 1% *Escherichia* Gut Abundance

Antibiotic	HR (95% CI)	P value
Beta-lactams	1.3 (0.5 – 3.1)	0.53
Fluoroquinolones	1.2 (0.4 – 3.3)	0.71
TMP-SMX PCP Prophylaxis	0.6 (0.2 - 1.8)	0.33
**Cefazolin Preoperative Prophylaxis**	**0.4 (0.2 – 0.9)**	**0.03**

Risk Factors for 1% *Enterococcus* Gut Abundance

Antibiotic	HR (95% CI)	P value
**Beta-lactams**	**4.6 (2.1 – 9.7)**	**1.3E-4**
Fluoroquinolones	2.1 (0.8 - 5.1)	0.11
TMP-SMX PCP Prophylaxis	1.8 (0.6 – 8.6)	0.35
Cefazolin Preoperative Prophylaxis	1.1 (0.5 – 2.6)	0.87

Antibiotics and Future Development of 1% Relative Gut Abundance of *Escherichia* and Future Development of 1% Relative Gut Abundance of *Enterococcus*. A Univariate Cox Proportion Hazard Model was created to assess the relationship between antibiotic use and future development of 1% relative gut abundance of *Escherichia* or *Enterococcus*. Antibiotics were grouped by class and were assessed as a time-dependent covariate for 1% relative gut abundance of *Escherichia* or *Enterococcus*. For each antibiotic class, the hazard ratio (HR) with 95% confidence intervals (CI) is reported with the associated *P* value. In bold text are the antibiotics that were associated with future development of 1% relative abundance of *Escherichia* or *Enterococcus* in univariate analysis. Abbreviations: HR: Hazard Ratio; TMP-SMX: trimethoprim/sulfamethoxazole; PCP: *Pneumocystis jiroveci*. Source data are provided as a source data file.

Supplementary Note 1

Metagenomic Sequencing of Cell Free DNA in the Urine Specimens and DNA in the Fecal Specimens

The supernatants from 17 urine specimens underwent single-stranded library preparation for cell-free DNA profiling. Shotgun metagenomics sequencing was performed with an average mean $\pm$ SD depth of 55 $\pm$ 8 million reads[1] with 75% $\pm$ 12% (mean $\pm$ SD) of sequences aligned to human chromosomes and removed. The mean amount of DNA obtained was 29 ng with a range of 10.5 ng to 146.9 ng per 1 mL of urine supernatant. Among the 14 urine specimens associated with *E. coli* bacteriuria, *E. coli* had an estimated mean 52X coverage of the *E. coli* genome with a range of 0.3X to 268X using 5,000,000 base pairs as the *E. coli* genome length. Among the 2 urine specimens associated with *E. faecalis* bacteriuria, *E. faecalis* had an estimated mean 10X coverage of the *E. faecalis* genome with a range of 7X to 14X using 3,000,000 base pairs as the *E. faecalis* genome length. Among the 2 urine specimens associated with *E. faecium* bacteriuria, *E. faecium* had an estimated mean 20X coverage of the *E. faecium* with a range of 1X to 39X using 3,000,000 base pairs as the *E. faecium* genome length.

The 17 paired fecal specimens underwent shotgun metagenomic sequencing with an average mean $\pm$ SD depth of 48 $\pm$ 6 million reads with 0.4% $\pm$ 0.9% (mean$\pm$ SD) sequences aligned to the human chromosomes and removed. Among the 14 fecal specimens associated with *E. coli* bacteriuria, *E. coli* had an estimated mean 100X coverage of the *E. coli* genome with a range of 13X to 371X using 5,000,000 base pairs as the *E. coli* genome length. Among the 2 fecal specimens associated with *E. faecalis* bacteriuria, *E. faecalis* had an estimated mean 108X coverage of the *E. faecalis* genome with a range of 5X to 211X using 3,000,000 base pairs as the *E. faecalis* genome length. Among the 2

fecal specimens associated with *E. faecium* bacteriuria, *E. faecium* had an estimated mean 396X coverage of the *E. faecium* genome with a range of 1X to 790X using 3,000,000 base pairs as the *E. faecium* genome length

Supplementary Figure 1

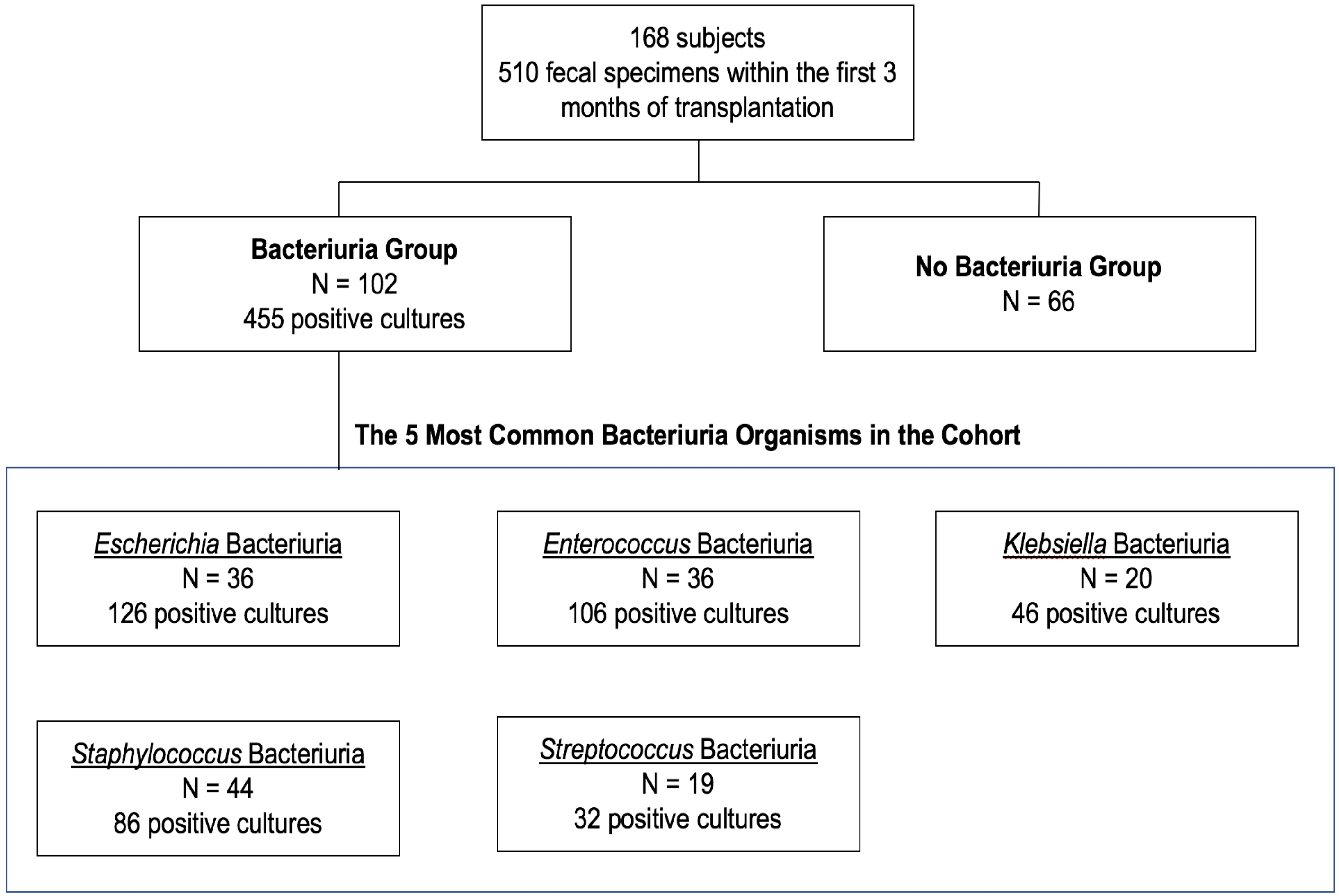

Study Cohort. Among the study cohort, 168 subjects provided 510 fecal specimens within the first 3 months of transplantation. One hundred two subjects developed bacteriuria and 66 subjects did not developed bacteriuria within the first 6 months of transplantation. The 5 most common genera associated with bacteriuria included *Escherichia, Enterococcus, Klebsiella, Staphylococcus,* and *Streptococcus.* Source data are provided as a source data file.

Supplementary Figure 2

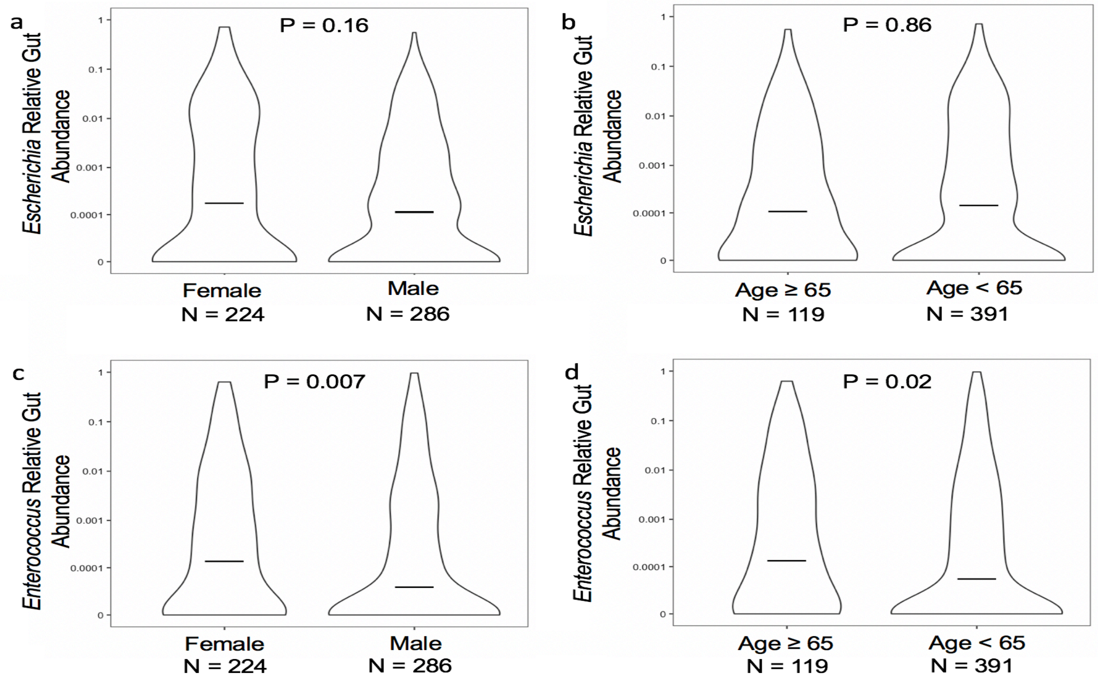

Relative Gut Abundance of *Escherichia* and *Enterococcus* by Gender and Age Group. (a) Relative gut abundance of *Escherichia* is on the y axis and gender is on the x axis. Violins plot are represented to reflect the distribution of data with the line within the violin plots representing the median. The relative gut abundance of *Escherichia* was not significantly different in the Female Group than in the Male Group (P=0.16, Wilcoxon rank sum test). **(b)** Relative gut abundance of *Escherichia* is on the y axis and Age ≥ 65 is on the x axis. Violins plot are represented to reflect the distribution of data with the line within the violin plots representing the median. The relative gut abundance of *Escherichia* was not significantly different in the Age ≥ 65 Group than in the Age < 65 Group (P=0.86, Wilcoxon rank sum test). **(c)** Relative gut abundance of *Enterococcus* is on the y axis and gender is on the x axis. Violins plot are represented to reflect the distribution of data with the line within the violin plots representing the median. The relative gut abundance of *Enterococcus* was significantly higher in the Female Group than in the Male Group (P=0.007, Wilcoxon rank sum test). **(d)** Relative gut abundance of *Enterococcus* is on the y axis and Age ≥ 65 is on the x axis. Violins plot are represented to reflect the distribution of data with the line within the violin plots representing the median. The relative gut abundance of *Enterococcus* was significantly higher in the Age ≥ 65 Group than in the Age < 65 Group (P=0.02, Wilcoxon rank sum test). Source data are provided as a source data file.

Supplementary Figure 3

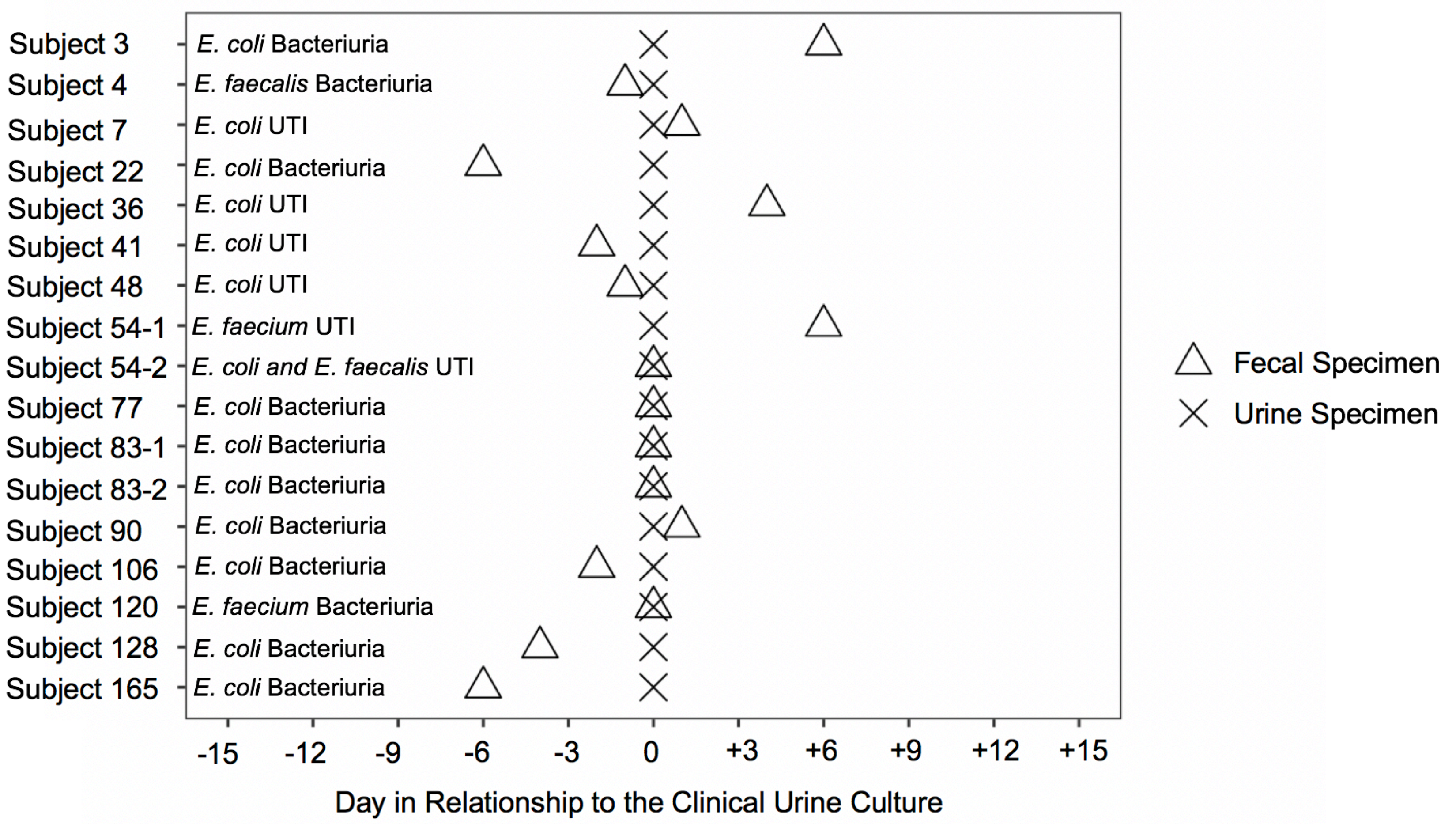

Timing of Collection of Fecal Specimens and Urine Specimens in the 17 Paired Cases for Strain Analysis. On the x axis is the day in relationship to when the clinical urine culture was obtained and on the y axis is the 17 cases for strain analysis. All urine specimens that underwent metagenomic sequencing were obtained on the same day as the urine culture, represented by an X shape. Fecal specimens that underwent metagenomic sequencing were obtained on the day represented by a triangle shape. The text inside the box on the left represents the clinical urine culture results as well as whether the urine culture was associated with bacteriuria or UTI. Source data are provided as a source data file.

Supplementary Figure 4

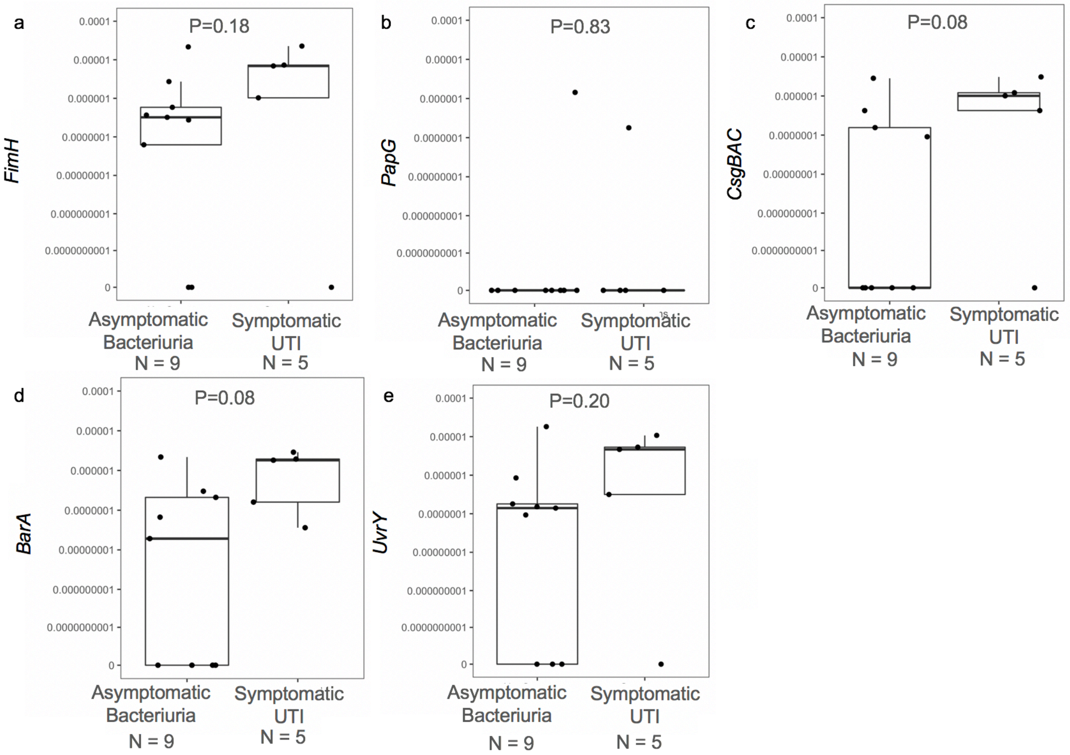

Uropathogenic Gene Relative Abundance in Asymptomatic *E. coli* Bacteriuria and Symptomatic *E. coli* UTI. In each graph, the relative abundance of uropathogenic genes is on the y axis and the status of asymptomatic *E. coli* bacteriuria or symptomatic *E. coli* UTI is on the x axis. Box and whisker plots are plotted with the median, 25% and 75% representing the edges of the boxplot,1.5 times the median representing the whiskers of the boxplot, and points representing individual specimens. All P values are calculated with the Wilcoxon rank sum test. Boxplots for a) *FimH* b) *PapG* c) *CsgBAC* d) *BarA* e) *UvrY*. Source data are provided as a source data file.

Supplementary Figure 5

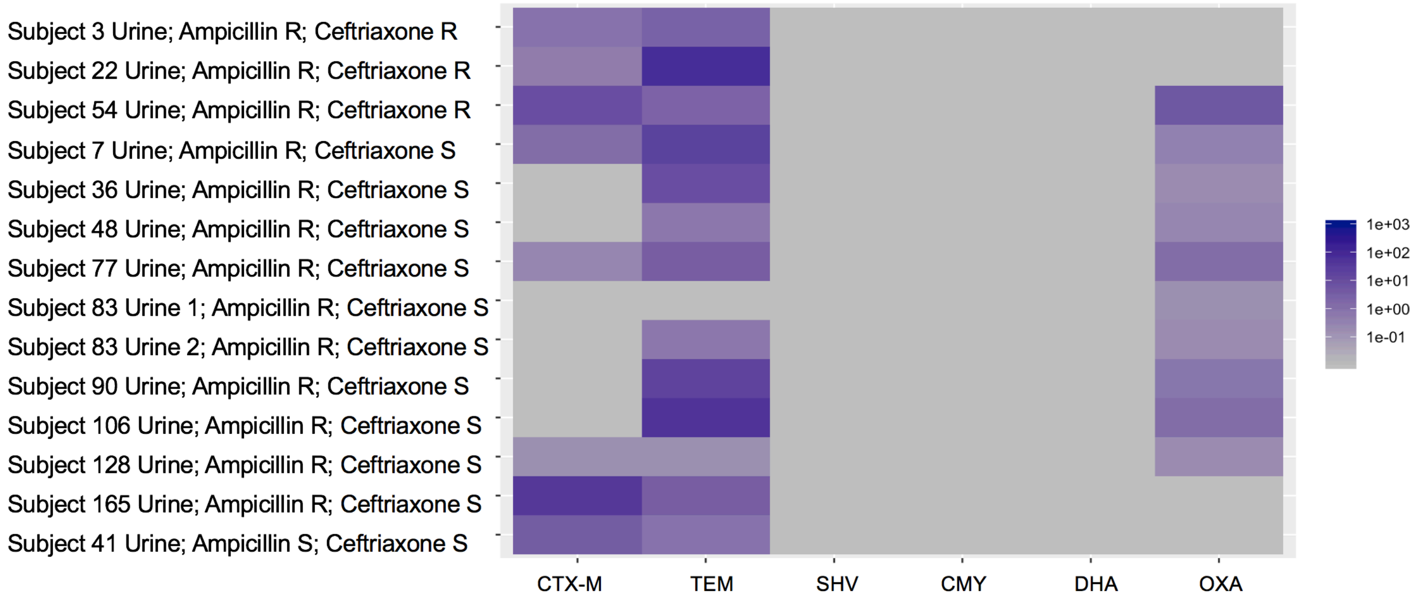

Beta-lactam Antibiotic Resistance Genes in *E. coli* Urine Samples. Antimicrobial resistance genes were determined using Bowtie2[2] on the MEGARES antibiotic resistance database[3] and RPKM was estimated for each of the following classes of beta-lactamase genes: CTX-M, TEM, SHV, CMY, DHA, and OXA. A heatmap was constructed with antibiotic resistance genes on the X axis and the urine specimens as well as the ampicillin and ceftriaxone antimicrobial susceptibility patterns of the *E. coli* isolated in culture on the Y axis. The abundance is colored by blue intensity, log scaled. Source data are provided as a source data file.

Supplementary References

1. Burnham P, *et al.* Urinary cell-free DNA is a versatile analyte for monitoring infections of the urinary tract. *Nat Commun* **9**, 2412 (2018).

2. Langmead B, Salzberg SL. Fast gapped-read alignment with Bowtie 2. *Nat Methods* **9**, 357-359 (2012).

3. Lakin SM, *et al.* MEGARes: an antimicrobial resistance database for high throughput sequencing. *Nucleic Acids Res* **45**, D574-D580 (2017).
