# Supplementary material for: Gut uropathogen abundance is a risk factor for development of bacteriuria and urinary tract infection
Source: Nat Commun. 2019 Dec 4;10:5521. doi: 10.1038/s41467-019-13467-w (PMC6893017; doi:10.1038/s41467-019-13467-w)
Supplement: Supplementary file 3 — Description of Additional Supplementary Files [file 41467_2019_13467_MOESM3_ESM.docx]

**Description of Additional Supplementary Files**

**File Name: Supplementary Data 1**

**Description:** Full list of genes/operons and the corresponding classes, derived from the MEGARES antibiotic genes database (Lakin et al., Nucleic Acids res 45, D574-D580, 2017). Version: megares_annotations_v1.01 (https://megares.meglab.org/download/index.php).
