# Supplementary material for: Gut uropathogen abundance is a risk factor for development of bacteriuria and urinary tract infection
Source: Nat Commun. 2019 Dec 4;10:5521. doi: 10.1038/s41467-019-13467-w (PMC6893017; doi:10.1038/s41467-019-13467-w)
Supplement: Supplementary file 5 — Reporting Summary [file 41467_2019_13467_MOESM5_ESM.pdf]

natureresearch

Corresponding author(s): John Lee

Last updated by author(s): Oct 30, 2019

Reporting Summary

Nature Research wishes to improve the reproducibility of the work that we publish. This form provides structure for consistency and transparency in reporting. For further information on Nature Research policies, see Authors & Referees and the Editorial Policy Checklist .

Statistics

For all statistical analyses, confirm that the following items are present in the figure legend, table legend, main text, or Methods section.

n/a	Confirmed	
☐	☒	The exact sample size (*n*) for each experimental group/condition, given as a discrete number and unit of measurement
☐	☒	A statement on whether measurements were taken from distinct samples or whether the same sample was measured repeatedly
☐	☒	The statistical test(s) used AND whether they are one- or two-sided<br>*Only common tests should be described solely by name; describe more complex techniques in the Methods section.*
☐	☒	A description of all covariates tested
☐	☒	A description of any assumptions or corrections, such as tests of normality and adjustment for multiple comparisons
☐	☒	A full description of the statistical parameters including central tendency (e.g. means) or other basic estimates (e.g. regression coefficient) AND variation (e.g. standard deviation) or associated estimates of uncertainty (e.g. confidence intervals)
☐	☒	For null hypothesis testing, the test statistic (e.g. *F*, *t*, *r*) with confidence intervals, effect sizes, degrees of freedom and *P* value noted<br>*Give P values as exact values whenever suitable.*
☒	☐	For Bayesian analysis, information on the choice of priors and Markov chain Monte Carlo settings
☒	☐	For hierarchical and complex designs, identification of the appropriate level for tests and full reporting of outcomes
☒	☐	Estimates of effect sizes (e.g. Cohen's *d*, Pearson's *r*), indicating how they were calculated

Our web collection on statistics for biologists contains articles on many of the points above.

Software and code

Policy information about availability of computer code

Data collection	No software was used
Data analysis	All analyses were performed in R 3.3.3 with packages yingtools2 0.0.0.89, phyloseq 1.19.1, dplyr 0.7.7, tibble 2.1.1, reshape2 1.4.3, data.table 1.10.4-3, scales 0.5.0, Hmisc 4.1-1, stringr 1.4.0, stringi 1.4.3, biomformat 1.2.0, deplyr 0.7.7, and coxphf 1.12, and ggtree 1.11.6. Bioinformatic processing of sequences were performed using kneaddata v.0.5.2, bowtie2 v0.1, MetaPhlAn2 2.6.0, StrainPhlAn 2.6.0, and HUMAnN2.

For manuscripts utilizing custom algorithms or software that are central to the research but not yet described in published literature, software must be made available to editors/reviewers. We strongly encourage code deposition in a community repository (e.g. GitHub). See the Nature Research guidelines for submitting code & software for further information.

Data

Policy information about availability of data

All manuscripts must include a data availability statement. This statement should provide the following information, where applicable:

- Accession codes, unique identifiers, or web links for publicly available datasets
- A list of figures that have associated raw data
- A description of any restrictions on data availability

All sequencing data as well as the deidentified clinical data that support the findings of this study will be made available before publication in the database of Genotypes and Phenotypes (dbGaP), accession number phs001879.v1.p1.
https://www.ncbi.nlm.nih.gov/projects/gapprev/gap/cgi-bin/study.cgi?study_id=phs001879.v1.p1
Local institutional review board approval will be needed to access the sequencing data as well as the deidentified clinical data.

Field-specific reporting

Please select the one below that is the best fit for your research. If you are not sure, read the appropriate sections before making your selection.

- [x] Life sciences
- [ ] Behavioural & social sciences
- [ ] Ecological, evolutionary & environmental sciences

For a reference copy of the document with all sections, see nature.com/documents/nr-reporting-summary-flat.pdf

Life sciences study design

All studies must disclose on these points even when the disclosure is negative.

Sample size	An a priori sample size determination was not performed given the limited preliminary data examining the relationship between gut microbial abundance and bacteriuria.
Data exclusions	While 280 kidney transplant recipients were consented into the protocol, only 168 kidney transplant recipients provided at least 1 fecal specimen and these 168 kidney transplant recipients were included in this study.
Replication	Fecal specimens were obtained if possible at post-transplant week 1, week 2, week 4, and week 12 as well as at time of urinary tract infections and post-transplant diarrhea with the hope that each subject would have multiple samples for profiling.
Randomization	This study was designed as a prospective cohort study and randomization was not performed. However, covariates were controlled for in multivariable cox regression analysis.
Blinding	The investigators performing the DNA extraction/16S rRNA sequencing were blinded to the UTI status of the kidney transplant recipients.

Reporting for specific materials, systems and methods

We require information from authors about some types of materials, experimental systems and methods used in many studies. Here, indicate whether each material, system or method listed is relevant to your study. If you are not sure if a list item applies to your research, read the appropriate section before selecting a response.

Materials & experimental systems

n/a	Involved in the study	
x		Antibodies
x		Eukaryotic cell lines
x		Palaeontology
x		Animals and other organisms
	x	Human research participants
x		Clinical data

Methods

n/a	Involved in the study	
x		ChIP-seq
x		Flow cytometry
x		MRI-based neuroimaging

Human research participants

Policy information about studies involving human research participants

Population characteristics	The population studied involved recruitment of kidney transplant recipients.
Recruitment	We recruited kidney transplant recipients at the time of transplantation to participate in urine, fecal, and blood specimen serially after kidney transplantation.
Ethics oversight	The Weill Cornell Medicine Institutional Review Board approved the study protocol.

Note that full information on the approval of the study protocol must also be provided in the manuscript.
