# Supplementary material for: Gut uropathogen abundance is a risk factor for development of bacteriuria and urinary tract infection
Source: Nat Commun. 2019 Dec 4;10:5521. doi: 10.1038/s41467-019-13467-w (PMC6893017; doi:10.1038/s41467-019-13467-w)
Supplement: Supplementary file 6 — Source Data [file 41467_2019_13467_MOESM6_ESM.zip › UTI_Code_Availability.zip!/README_UTI_092319.docx]

**R Script for Generating Phylogenetic Tree Plot**

Necessary Software Installation

R : <https://www.r-project.org/>

R studio : <https://www.rstudio.com/>

Versions Used During Demo Analysis

R 3.4.4 ; Rstudio 1.1.447

Necessary R Packages Installation

Using the install_packages command, install ggtree and ggplot2

Versions Used During Demo Analysis

ggtree 1.11.6, ggplot2 2.2.1

Typical installation time: < 30 minutes

After installation of R and R studio, load R script titled UTI_GGTree.R and set working directory with the files: RAxML_bestTree.s__Escherichia_coli.tree (RAxML_Ecoli tree source data for Fig 2a) and metafile_Ecoli_071919.txt (metafile Ecoli source data for Fig 2a)

Run R script

Expected output: Test_Tree tiff file, which is Fig. 2a in the manuscript. (also, Expected test output from custom R script for producing phylogenetic tree graph)

Run time: < 5 minutes

This R script can be run on any RAxML_bestTree output from the StrainPhlAn analysis

**Python Script for Assigning Taxonomy**

Necessary software installations:

Blast: <https://blast.ncbi.nlm.nih.gov/Blast.cgi?PAGE_TYPE=BlastDocs&DOC_TYPE=Download>

Python: <https://www.python.org>/

Biopython: <https://biopython.org/>

Versions Used During Demo Analysis

BLAST 2.7.1+

Python 2.7.6

Biopython 1.63

Installation time: < 30 minutes

Download taxonomic databases/nodes

<ftp://ftip.ncbi.nlm.nih/gov/pub/taxonomy/taxdump.tar.gz>

Important files include: names.dmp, nodes.dmp, and merged.dmp. Specify location as per python script.

Download Blast RefSeq_RNA databases or can update via script (see python script):

<ftp://ftp.ncbi.nlm.nih.gov/blast/db/>

After installation of stand alone Blast, Python, and Biopython and downloading taxonomic databases and Refseq_RNA databases, make the blast directory as part of the environment variables.

The demo file is: test.fasta (with 5 sequences) (test FASTA file for custom python script for assigning taxonomy)

Run in the command line:

python blastn_standalone.py test.fasta (custom python script for assigning taxonomy)

The output should be:

Test.fasta.blastn.refseq_rna.txt (Expected test output 1 from custom python script for assigning taxonomy)

Test.fasta.blastn.refseq_rna.txt.log (Expected test output 2 from custom python script for assigning taxonomy)

Run time < 5 minutes

This python script can be run on any fasta file to assign taxonomy
